# PKC Activation by Resveratrol Derivatives with Unsaturated Aliphatic Chain

**DOI:** 10.1371/journal.pone.0052888

**Published:** 2012-12-21

**Authors:** Satyabrata Pany, Anjoy Majhi, Joydip Das

**Affiliations:** Department of Pharmacological and Pharmaceutical Sciences, College of Pharmacy, University of Houston, Houston, Texas, United States of America; University of Iowa, United States of America

## Abstract

Resveratrol (**1**) is a naturally occurring phytoalexin that affects a variety of human disease models, including cardio- and neuroprotection, immune regulation, and cancer chemoprevention. One of the possible mechanisms by which resveratrol affects these disease states is by affecting the cellular signaling network involving protein kinase C (PKC). PKC is the family of serine/threonine kinases, whose activity is inhibited by resveratrol. To develop PKC isotype selective molecules on the resveratrol scaffold, several analogs (**2–5**) of resveratrol with a long aliphatic chain varying with number of unsaturated doubled bonds have been synthesized, their cytotoxic effects on CHO-K1 cells are measured and their effects on the membrane translocation properties of PKCα and PKCε have been determined. The analogs showed less cytotoxic effects on CHO-K1 cells. Analog **4** with three unsaturated double bonds in its aliphatic chain activated PKCα, but not PKCε. Analog **4** also activated ERK1/2, the downstream proteins in the PKC signaling pathway. Resveratrol analogs **2–5**, however, did not show any inhibition of the phorbol ester-induced membrane translocation for either PKCα or PKCε. Molecular docking of **4** into the activator binding site of PKCα revealed that the resveratrol moiety formed hydrogen bonds with the activator binding residues and the aliphatic chain capped the activator binding loops making its surface hydrophobic to facilitate its interaction with the plasma membrane. The present study shows that subtle changes in the resveratrol structure can have profound impact on the translocation properties of PKCs. Therefore, resveratrol scaffold can be used to develop PKC selective modulators for regulating associated disease states.

## Introduction

**Figure 1:**
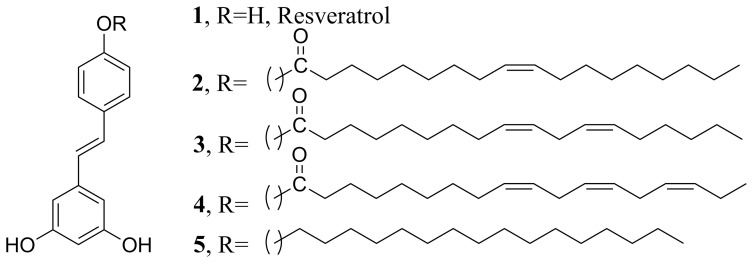
Chemical structure of compounds 1–5.

Resveratrol (**1**, Figure 1) is a naturally occurring phytoalexin found in grapes, red wine, peanuts, olive oil, cranberries, and other food [1], [2]. Numerous studies highlighted the effects of resveratrol in a variety of human disease models, including cardio- and neuroprotection, immune regulation, and cancer chemoprevention [3]–[8]. Some of the recent studies evaluated its promising biological properties, [9], [10] including antioxidant activity, [11] antiestrogenic activity [12] inhibition of cyclooxygenase [13] and inhibition of platelet aggregation [14]. Resveratrol showed chemopreventive activities against human degenerative diseases such as atherosclerosis [15] and cancer [16]. Resveratrol showed cancer chemopreventive activity in assays representing anti-initiation, anti-promotion and antiprogression activity, inhibiting the development of preneoplastic lesions and tumorigenesis [16]. Further evidence showed that it inhibits cell growth and induces apoptosis in various human cancer cell lines [17]–[21]. In particular, a number of studies reported the chemopreventive activity of resveratrol against prostate cancer [22]–[24]. Resveratrol is also currently in clinical phase II trials as an anti-cancer drug for treatment of human colon cancer [25], [26].

Resveratrol displays its biological response by acting on multiple targets. The activity of resveratrol has been linked to cell-surface receptors, membrane signaling pathways, intracellular signal-transduction machinery, nuclear receptors, gene transcription, and metabolic pathways [27], [28]. Protein kinase C (PKC) is one of the many targets of resveratrol.

PKC [29]–[31] belongs to the family of serine/threonine kinases involved in the regulation of various aspects of cell functions, including cell growth, differentiation, metabolism, and apoptosis [32]. PKC's role has been implicated in the pathology of several diseases such as cancer, diabetes, stroke, heart failure, and Alzheimer's disease [33]–[39]. PKC has been a subject of intensive research and drug development in the area of cancer [40].

The PKC family has been divided into three main groups: conventional isoforms (α, βI, βII and γ) that require Ca^2+^ and diacylglycerol (DAG) for activation; novel isoforms (δ, ε, η, θ and µ) that require only DAG and atypical isoforms (ζ, ι and λ) that require neither Ca^2+^ nor DAG [41]. The conventional and novel PKCs have four domains, termed C1 through C4, that play distinct roles in kinases' function. C1 and C2 are regulatory domains, C3 is the ATP binding domain, and C4 is the catalytic domain. DAG contains two long chains, acts as a second messenger [42] by binding to the C1 domain and inducing the translocation of PKCs to discrete subcellular compartments. Phorbol esters, which are isolated from plants, activate PKCs several fold higher than DAG by binding to the C1 domain. In the conventional and novel PKC isoenzymes, the DAG-sensitive C1 domain is duplicated into a tandem C1 domain consisting of C1A and C1B subdomains. The C1 domains have become an attractive target in designing the PKC based drugs. Recently, it has been found that alcohol and anesthetics also bind to the PKC C1 domains [43]–[45].

The biological effects of resveratrol on PKCs have been studied both in the cellular system and in *in vitro* purified proteins. Resveratrol regulates cellular PKCα and PKCδ to inhibit growth and induce apoptosis in gastric cancer cells [46]. It also inhibits cyclooxygenase-2 transcription and activity in phorbol ester-treated human mammary epithelial cells [47] and antagonizes EGFR-dependent Erk1/2 activation in human androgen-independent prostate cancer cells with associated isozyme-selective PKCα inhibition [48]. Resveratrol also preferentially inhibits PKC-catalyzed phosphorylation of a cofactor-independent, arginine-rich protein substrate by a novel mechanism [49].

The mechanism of the effects of resveratrol on the activities of purified recombinant PKC isozymes induced by association with model lipid vesicle membranes was investigated using an in vitro assay system in which the cofactor and activator-concentration dependencies for activation were systematically varied [50]. It was found that resveratrol inhibited membrane-associated PKCα activity within a concentration range relevant to the cellular effects of the stilbene [11], [16], [51]–[53] and it was proposed that resveratrol binds to the C1 domain of PKCα [50]. In a previous study, we measured the effect of several resveratrol derivatives on PKCα activity in HEK293 cells [54].

In the present study, we describe the synthesis of several resveratrol derivatives having unsaturated aliphatic chain and their effects on the translocation properties of PKCα and PKCε in the presence and absence of a phorbol ester, 12-O-tetradecanoylphorbol-13-acetate (TPA). Our results show that chemical modification of one of the hydroxyl groups of resveratrol with aliphatic carbon chain reduced its cytotoxicity on CHO-K1 cells. Modification with a linolenyl chain completely abolished resveratrol's inhibitory effects on PKC. Instead, the molecule activated PKCα and the downstream protein ERK1/2 in the PKC signaling pathway.

## Results

### Absorption and emission spectra of resveratrol (1) and its derivatives (2–5)

**Figure 2:**
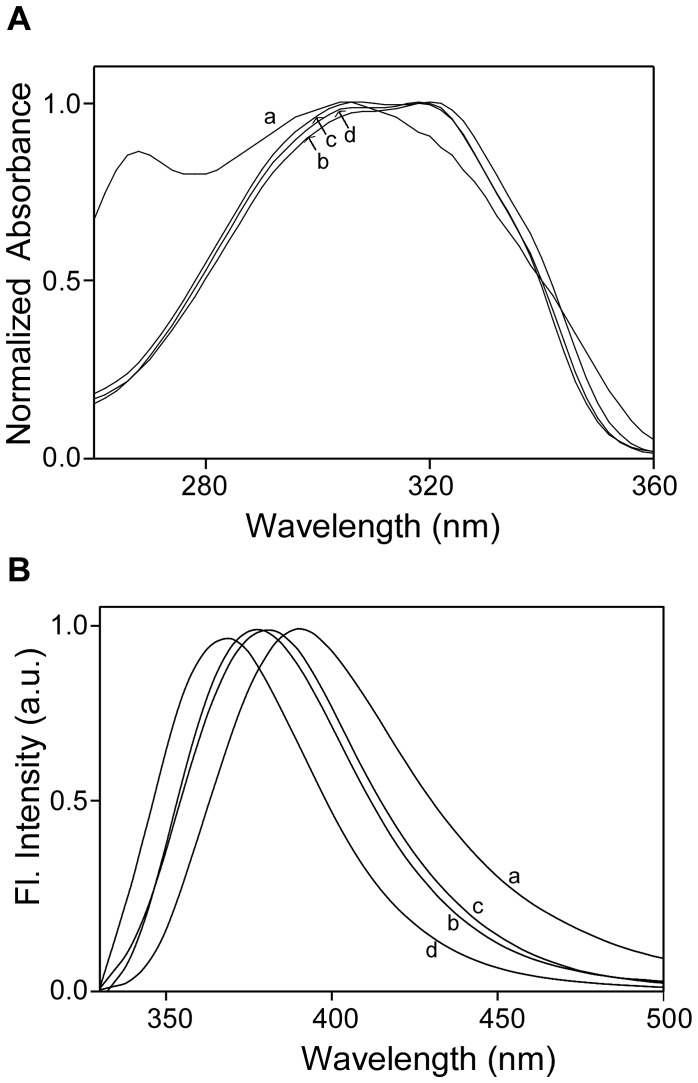
Effect of solvent polarity on the absorption and fluorescence properties of 2 (5–10 µM). A), Normalized absorption and B) normalized florescence emission spectra of **2** in a) water, b) ethanol, c) acetonitrile and d) hexane.

The absorption and emission maxima of resveratrol (**1**) and its derivatives (**2–5**) in various organic solvents are listed in Table 1. Figure 2A shows the representative absorption spectra of **2** in different solvent. Resveratrol (**1**) showed broad absorption maxima in the range of 304–318 nm with two humps at around 306 nm and 319 nm. This band did not show any significant wavelength shift when the solvent was switched from polar ethanol to nonpolar hexane. For the resveratrol derivatives **2–4**, absorbance of the 319 nm band was slightly higher than the 306 nm band in ethanol and acetonitrile and the absorbance of the 306 nm band was higher than the 319 nm band in hexane and water. For compound **5,** the 306 nm band was higher than the 319 nm bands in ethanol, acetonitrile and hexane, whereas in water it showed a 275 nm band.

**Table 1:** Absorption and fluorescence maxima of resveratrol (1) and its derivatives (2–5) in different solvent at 25°C.

Compound	Absorbance maximum (λ_max_), nm				Emission maximum (λ_em_), nm			
	Ethanol	Acetonitrile	Hexane	Water	Ethanol	Acetonitrile	Hexane	Water
**1**	306	305	306	305	379	377	373	392
**2**	320	319	307	305	374	379	366	389
**3**	320	319	307	305	379	384	365	387
**4**	320	319	308	305	379	381	365	392
**5**	307	306	306	275	379	378	368	386

The emission maximum of resveratrol is in the range of 373–379 nm in organic solvents, whereas it is red shifted to 392 nm in water. Similar to the emission characteristics of resveratrol, the derivatives **1–5** also showed highest emission maxima values in water and the lowest emission maximum values in hexane (Figure 2B). There was no significant difference in the emission maxima values of **2**, **3** and **4,** in which the number of unsaturated double bonds were different.

Overall, the chemical modification of resveratrol in **2–5** did not show any significant changes in the absorption and emission characteristics of resveratrol.

### Effect of 1–5 on cell viability

**Figure 3:**
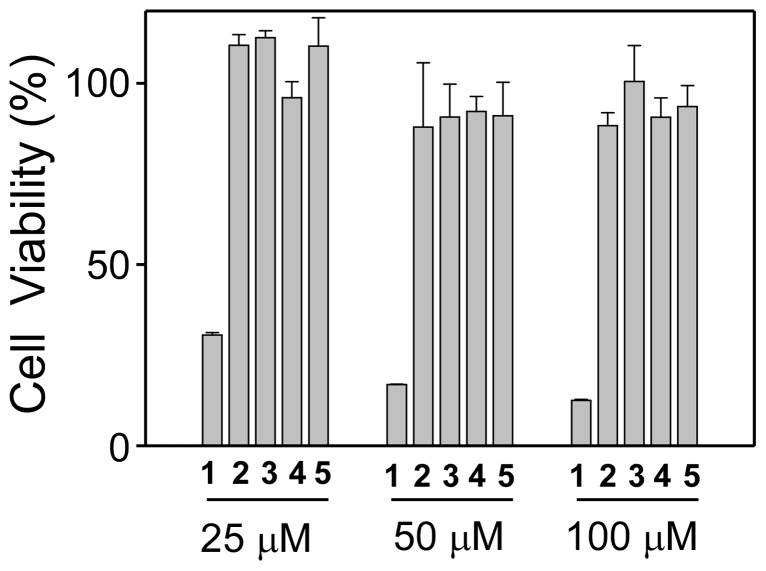
Effect of 1–5 on CHO-K1 cell viability. The graph shows the percentage of viable cell after treatment with **1–5** at three different concentrations for 48 h. The cell viability was measured by MTT assay. Mean and standard deviation (SD) were obtained from three experiments done in triplicate.

Resveratrol is known to exert toxic effects on different cell lines [55]. Results shown in Figure 3 indicated that resveratrol produced a marked, concentration dependent reduction in number of viable CHO-K1 cells. For resveratrol, ∼30% cell viability was observed at 25 µM and ∼ 15% cell viability was observed at 100 µM as compared to the untreated control cells. In contrast, for compounds **2–5** cell viability was much higher as compared to resveratrol. Additionally, no significant differences were observed among **2–5**, which contained different number of unsaturated double bonds. In conclusion, modification of resveratrol with unsaturated hydrocarbon chain significantly reduced the cytotoxicity of resveratrol.

After evaluating the cytotoxic property of resveratrol and its derivatives, we studied the effect of these derivatives on expression, activation and inhibition of PKCα and PKCε.

### Effect of 1–5 on PKCα and PKCε expression

**Figure 4:**
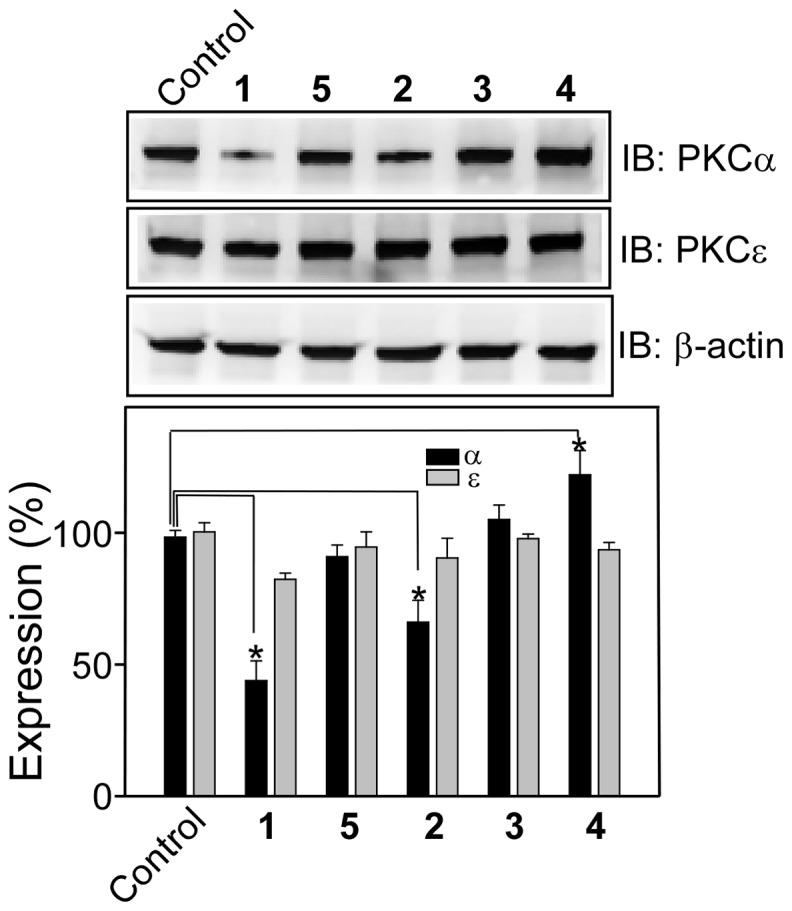
Effect of 1–5 on the expression of PKCα and PKCε. Upper panels, Western blot analysis of whole cell lysate of CHO-K1 cells after treatment with **1–5** (10 µM) for 24 h. Lower panel, bar graph of densitometry analysis of PKC expression (Mean ± SE, *P<0.05, n = 3). β actin was used as a reference for uniform loading. Control refers to the sample with no addition of compounds.

Effect of **1–5** on the expression of PKCα and PKCε was examined by immunoblot methods using the whole cell lysate of CHO-K1 cells treated with the compounds (10 µM) for 24 h. Results indicated that PKCα expression was significantly reduced (∼60%) when the cells were treated with **1** as compared to the untreated cells (Figure 4). About 30% decrease in PKCα expression was observed for **2**. In contrast, **4** showed increase in expression, but **3** and **5** did not show any effect on PKCα expression. Moreover, none of these compounds showed any effect on the expression of PKCε. To summarize, no particular trend is observed on their effects on PKCα expression, however compound **4** with three unsaturated double bonds increased the expression of PKCα, but not of PKCε in CHO-K1 cells.

### Effect of 1–5 on membrane translocation of PKCα and PKCε

**Figure 5:**
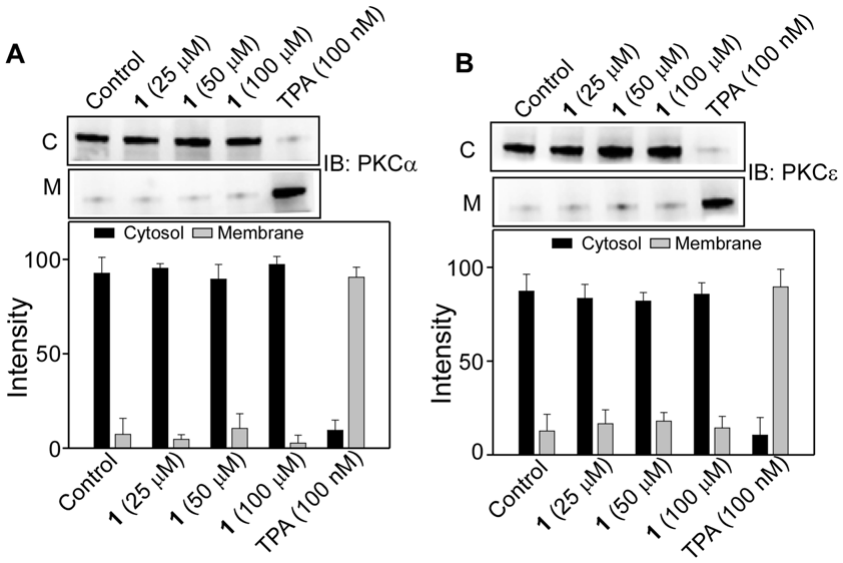
Effect of 1 on the membrane translocation of PKCα and PKCε. Upper panels, Western blot analysis of the cytosolic (C) and the membrane (M) fractions of (**A**) PKCα and (**B**) PKCε after the cells were treated with varying concentration **1** for 1 h. Lower panel, bar graph of densitometry analysis of the upper panel immunoblots (Mean± SE, n = 3). Control refers to the sample with no addition of compounds.

**Figure 6:**
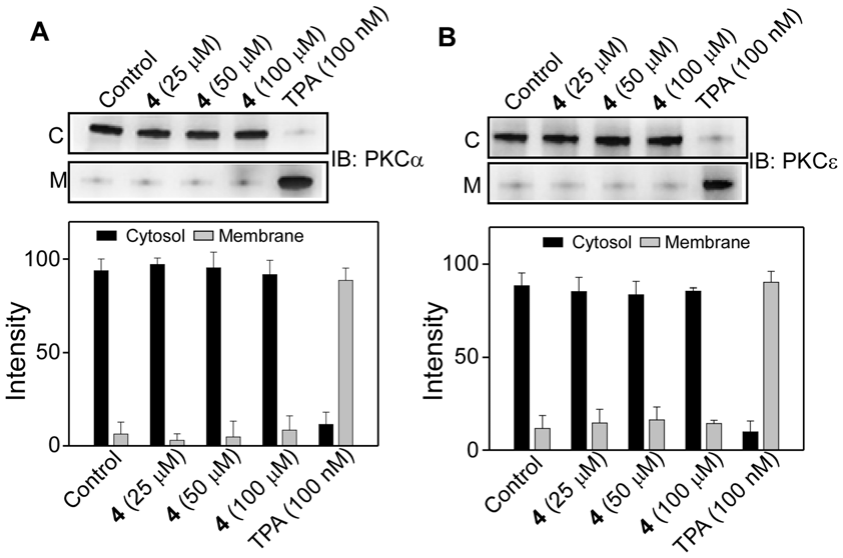
Effect of 4 on the membrane translocation of PKCα and PKCε. Upper panels, Western blot analysis of the cytosolic (C) and the membrane (M) fractions of (**A**) PKCα and (**B**) PKCε after the cells were treated with varying concentration of **4** for 1 h. Lower panel, bar graph depicts the densitometry analysis of the upper panel immunoblots (Mean± SE, n = 3). Control refers to the sample with no addition of compounds.

**Figure 7:**
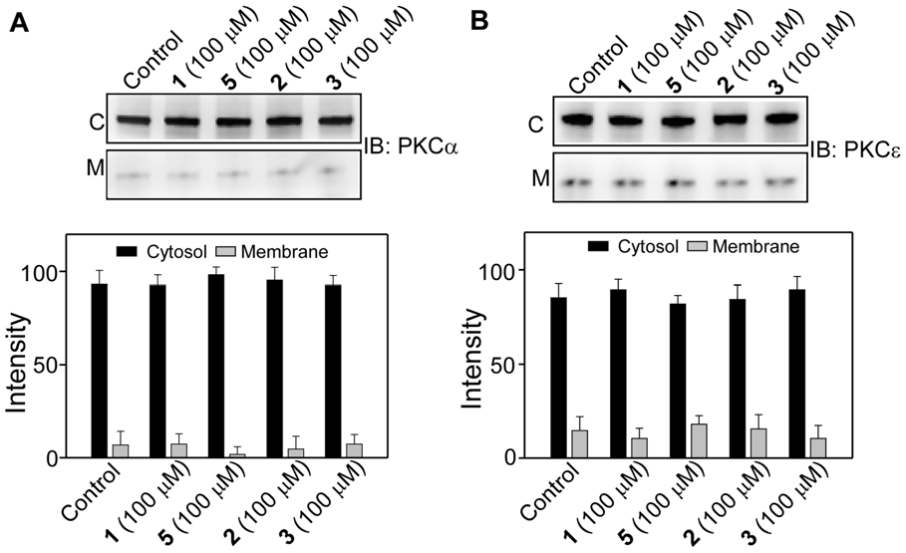
Effect of 1, 2, 3, and 5 on the membrane translocation of PKCα and PKCε. Upper panels, Western blot analysis of the cytosolic (C) and the membrane (M) fractions of (**A**) PKCα and (**B**) PKCε after the cells were treated with 100 µM of **2, 3** or **5** for 1 h. Lower panel, bar graph of densitometry analysis of the upper panel immunoblots (Mean± SE, n = 3). Control refers to the sample with no addition of compounds.

Effects of **1–5** on the translocation properties of PKCα and PKCε were measured at two different incubation times, 1 h and 24 h. Incubation of resveratrol (**1**) at 25–100 µM for 1 h with either PKCα or PKCε did not show any PKC membrane translocation. Under the similar condition however, 100 nM TPA caused almost complete membrane translocation of both PKCα and PKCε (Figure 5). For compounds **2, 3, 4** and **5** also, no translocation of either PKCα or PKCε was observed (Figures 6 and 7).

**Figure 8:**
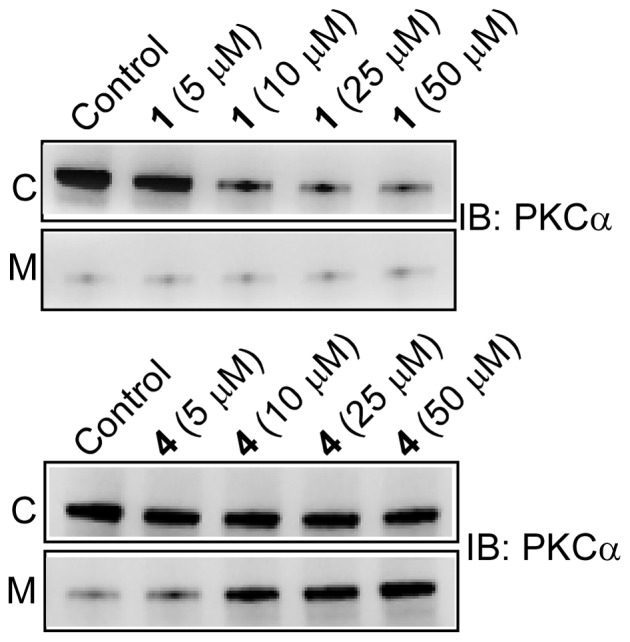
Effect of 1 and 4 on the membrane translocation of PKCα. Western blot analysis of the cytosolic (C) and the membrane (M) fractions of PKCα after the cells were treated with varying concentration of **1** and **4** for 24 h. Control refers to the sample with no addition of compounds.

**Figure 9:**
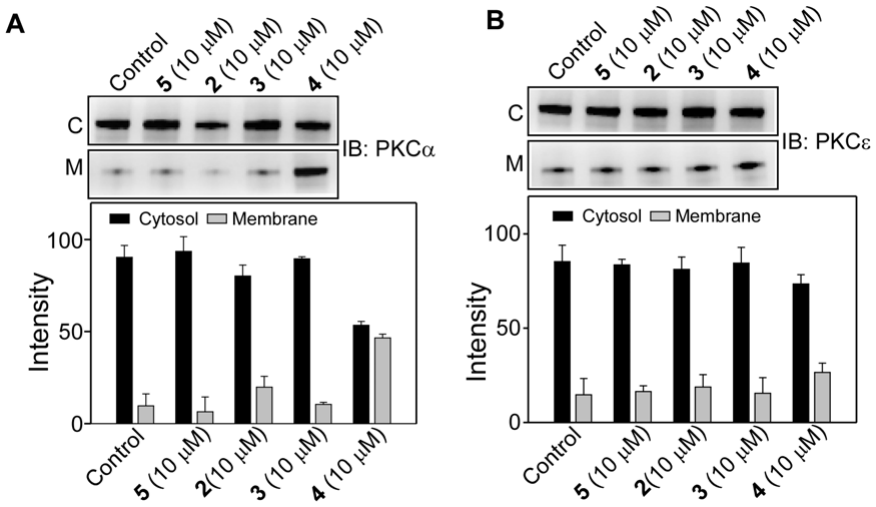
Effect of 2–5 on the membrane translocation of PKCα and PKCε. Upper panels, Western blot analysis of the cytosolic (C) and the membrane (M) fractions of (**A**) PKCα and (**B**) PKCε after the cells were treated with 10 µM of **2, 3, 4** or **5** for 24 h. Lower panel, bar graph of densitometry analysis of the upper panel immunoblots (Mean± SE, n = 3). Control refers to the sample with no addition of compounds.

Next, we examined the effects of **1–5** on membrane translocation of PKCα and PKCε with an incubation time of 24 h. Figure 8 shows the distribution of PKCα in cytosol and membrane after cells were treated with varying concentration of **1** and **4**. Results indicated that cytosolic PKCα level decreased with increasing concentration of **1,** and there was no visible effect on the membrane fraction. In contrast, **4** increased the PKCα level in membrane and its level in cytoplasm remained unchanged. These observations are correlated well with our earlier observation that compound **1** decreased the expression of PKCα and compound **4** increased it. For compound **4**, membrane PKCα level considerably increased at the concentration range of 5–10 µM and at concentration higher than 10 µM, this increment was rather small. Therefore, the concentration 10 µM was chosen to study the membrane translocation properties of all the compounds. Figure 9 clearly indicated that compound **4** was able to increase the amount of PKCα in the membrane. However, when cells were treated with 10 µM of **2**, **3** or **5** for 24 h, no significant effect on the cytosolic and membrane fraction of PKCα was observed (Fig. 9). Additionally, cells treated with 10 µM of **1–5** also did not show any effect on PKCε. Overall, among all the resveratrol derivatives tested on PKCα and PKCε, **4** is the only compound that increased the amount of PKCα in the membrane at 24 h incubation.

To confirm whether the increase of PKCα in the membrane fraction was due to PKCα translocation from cytosol to membrane, we examined the effect of **4** on the activation ERK, the downstream signaling cascade molecule activated by membrane-translocated and activated PKCα.

### Effect of 3 and 4 on ERK1/2 phosphorylation

**Figure 10:**
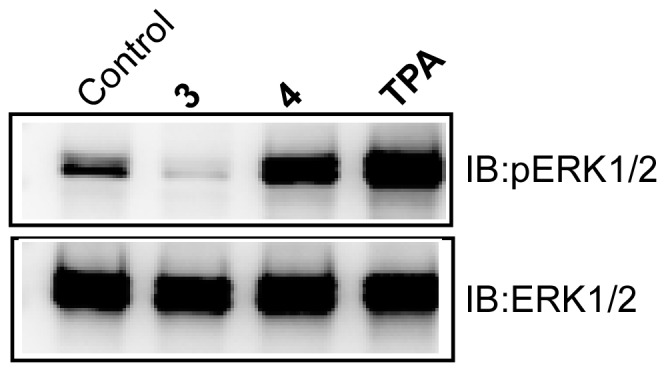
Effect of 3 and 4 on ERK1/2 phosphorylation in CHO-K1 cells. Cells were either treated with 10 µM of **3** or **4** for 24 h or 100 nM of TPA for 1 h for Western blot analysis. Control refers to the sample with no addition of compounds.

To confirm that the membrane translocation, and the activation of PKCα by **4**, is propagated along the signal transduction pathway, the effect of **4** on the activation of the downstream ERK1/2 was undertaken. Activation of ERK1/2 was determined by the extent of its phosphorylation in response to **4**. Compound **4** phosphorylated ERK1/2, whereas **3** did not do so (Figure 10). For TPA, as expected, higher extent of phosphorylation was observed in 1 h. The conclusion is that activation of PKCα by **4** is transduced along the signal transduction pathway.

### Effect of oleic acid, linoleic acid and linolenic acid on the membrane translocation of PKCα and PKCε

**Figure 11:**
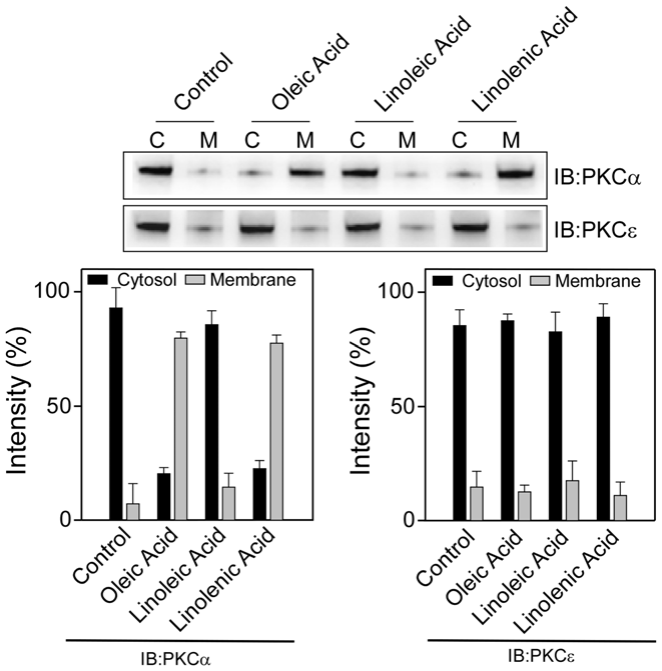
Effect of oleic acid, linoleic acid and linolenic acid on the membrane translocation of PKCα and PKCε. Upper panels, Western blot analysis of the cytosolic (C) and the membrane (M) fraction of (**A**) PKCα and (**B**) PKCε after the cells were treated with 10 µM of oleic acid, linoleic acid or linolenic acid for 24 h. Lower panel, bar graph of densitometry analysis of upper panel immunoblots (Mean± SE, n = 3). Control refers to the sample with no addition of compounds.

To determine if the unsaturated hydrocarbon chains of **2–4** play any role in the membrane translocation properties of PKCα and PKCε, we examined the effect of oleic acid, linoleic acid and linolenic acid on the membrane translocation of PKCα and PKCε. When CHO-K1 cells were treated separately with 10 µM of oleic acid, linoleic acid and linolenic acid for 24 h, no particular trend was observed in the membrane translocation for these three fatty acids. Oleic acid and linolenic acid both showed about 75% membrane translocation of PKCα from cytosol to membrane (Figure 11). However no effect on PKCα was observed for linoleic acid at this concentration. Furthermore, none of the compounds showed any effect on membrane translocation of PKCε. In conclusion, oleic acid and linolenic acid, which contain one and three unsaturated double bonds respectively, caused the membrane translocation of PKCα.

A previous study on the effect of fatty acids on PKC translocation in CHO-K1 revealed that oleic acid and linolenic acid translocated a conventional PKC, PKCγ to the membrane, whereas linoleic acid translocated it to the perinuclear membrane. These three fatty acids however did not show significant effect on PKCε [56].

### Effects of 1–5 on TPA-induced membrane translocation of PKCα and PKCε

**Figure 12:**
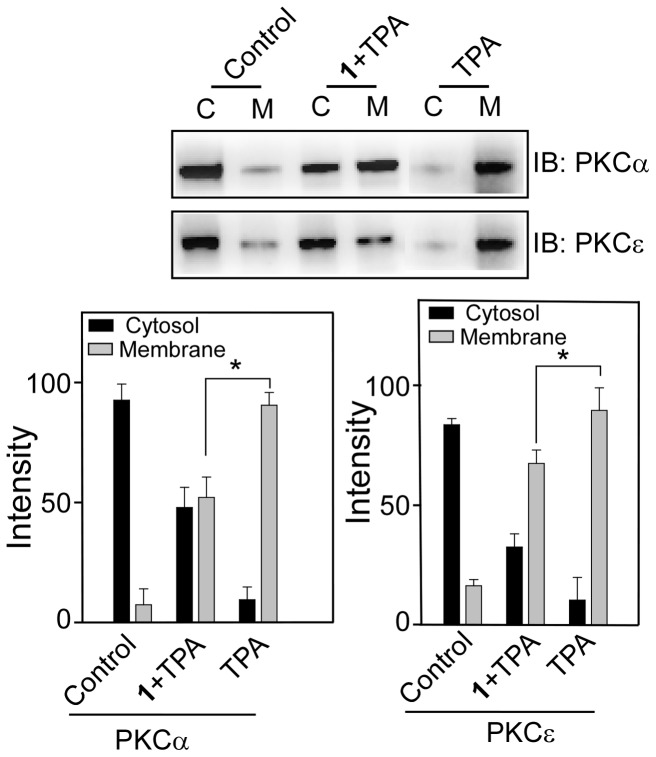
Effect of 1 on TPA-induced membrane translocation of PKCα and PKCε. Upper panels, Western blot analysis of the cytosolic (C) and the membrane (M) fraction of (**A**) PKCα and (**B**) PKCε. Lower panel, bar graph of densitometry analysis of upper panel immunoblots (Mean ± SE, *P<0.05, n = 3). Cells were treated with 100 µM of **1** in the presence and absence of 100 nM TPA for 1 h. Control refers to the sample with no addition of compounds.

**Figure 13:**
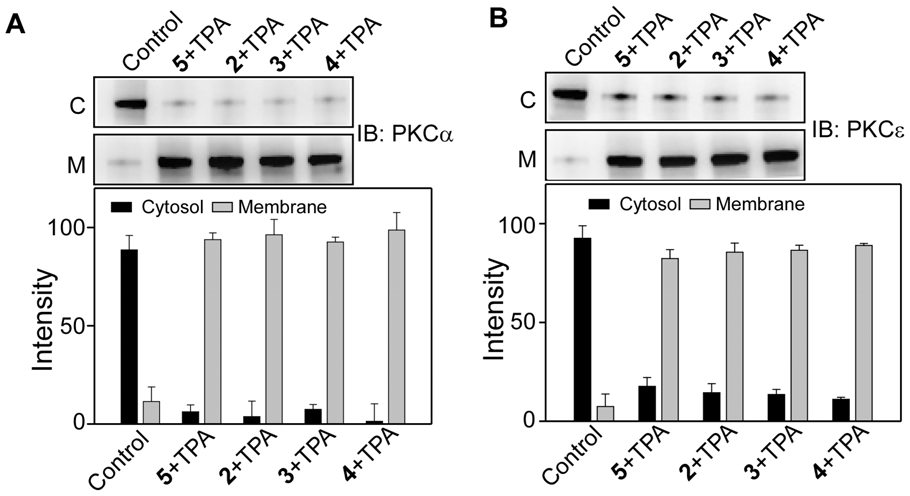
Effect of 2–5 on TPA-induced membrane translocation of PKCα and PKCε. Upper panels, Western blot analysis of the cytosolic (C) and membrane (M) fraction of (**A**) PKCα and (**B**) PKCε. Lower panel, bar graph of densitometry analysis of upper panel immunoblots (Mean ± SE, n = 3). Cells were treated with 100 µM of **2–5** and 100 nM of TPA for 1 h. Control refers to the sample with no addition of compounds.

Studies with both purified and cellular PKCs indicated that resveratrol is a PKC inhibitor and this inhibitory property is isoform specific [50], [57]. To investigate if chemical modification of resveratrol could affect the inhibitory properties, we examined the effect of **1–5** on the TPA-induced membrane translocation of PKCα and PKCε. When CHO-K1 cells were co-treated with **1–5 **(100 µM) and TPA (100 nM) for 1 h, there was ∼45% and ∼20% reductions in the TPA-induced membrane translocation of PKCα and PKCε, respectively (Fig. 12). In contrast, under similar experimental condition, **2–5** did not show any effect on TPA-induced translocation of PKCα and PKCε, as majority of PKC was localized in the membrane, similar to when the cells were treated with TPA alone (Fig. 13). This means that chemical modification of resveratrol with long chains resulted in the complete loss of the inhibitory properties of resveratrol towards both PKCα and PKCε.

## Discussion

The present study represents our ongoing effort of developing isoform selective PKC regulator using simpler chemical scaffolds. In a previous study [54] we demonstrated that resveratrol moiety, that possesses the hydroxyl pharmacophore of the PKC activator phorbol esters, modulated PKCα activity. In the present study, a series of long chain derivatives of resveratrol with varying degree of unsaturation in the aliphatic chain have been synthesized and their abilities to activate and inhibit PKCα and PKCε have been tested. PKCα and PKCε belong to the conventional and novel type respectively. Unlike our previous study in which the PKCα was overexpressed in HEK293 cells, [54] in the present study we used CHO-K1 cells where both PKCα and PKCε are endogenously expressed. The rationale for studying the unsaturated long chains is that both phorbol ester and diacylglycerol contain long aliphatic chain with the latter having unsaturation in the chain. An earlier report showed that addition of a long aliphatic chain to the indolactam moiety enhanced PKC activity [58].

We have thoroughly investigated the activity of PKCα and PKCε by measuring their translocation to the plasma membrane in response to the synthetic derivatives. We observed increase in PKCα in the membrane fraction while the amount in the cytosolic fraction remained similar to the control (panel A of figure 9). The increase in membrane PKCα could be due the increased protein synthesis and concomitant membrane translocation, the extent of which was not quantitatively measured in the present study. However, observed activation of ERK1/2 by **4** strongly suggested that **4** caused membrane translocation of PKCα thereby activating ERK1/2. The major finding of our study is that the modification of the resveratrol moiety reduced cytotoxicity significantly, and compound **4** with three unsaturated double bonds in its aliphatic chain (18∶3), activated PKCα. This property of **4** is quite different from resveratrol which did not activate but inhibited the stimulated activity of PKCα. All the molecules were inert towards PKCε both in the activation and inhibition processes. For resveratrol, we detected 20% inhibition for PKCε and 45% inhibition for PKCα. While our data is consistent with the inhibition data reported earlier for PKCα in PC-3 cells, our PKCε inhibition data is not in agreement with earlier study in PC-3 cell in which the authors reported no inhibition of PKCε. Contrasting results were however reported with purified protein. While Stewart et al [49] reported that resveratrol inhibited both PKCα and PKCε, Slater et al [50] reported inhibition of only PKCα, not the PKCε. These discrepancies reflect the differences in the machinery present in different cell lines and variety of lipid mixture and cofactor used in the vitro assay systems.

The resveratrol derivatives were synthesized by combining two moieties, the resveratrol moiety and the fatty acyl chain. While previous studies showed that resveratrol did not activate, but inhibited the phorbol ester induced activation, among all the compounds tested in this study only compound **4** showed activation of PKCα. To find out why compound **4** activated PKCα while the parent resveratrol inhibited PKCα, we did control experiments with the fatty acid component of the structures, the oleic acid, linoleic acid and linolenic acid. Several studies were reported on the effects of fatty acids on PKC activities, showing both activation [56], [59]–[63] and inhibition, [64] extent of which depended on the cell type, number and position of the double bonds, state of the protein-whether purified or in cells etc. Our study is closely resembled with the study reported in the CHO-K1 cell line by Shirai et al [56]. In this study both oleic acid and linolenic acid translocated a conventional PKC, PKCγ to the membrane but showed little effect on the novel PKCε. Linoleic acid, on the other hand, translocated PKCγ to the perinuclear region. This is more or less consistent with our results in that PKCε is insensitive towards these fatty acids and our results for PKCα are similar to PKCγ both of which belong to the conventional class of PKC. It is possible that we could not detect the effect of linoleic acid because we measured the translocation of the protein to the membrane, not to the perinuclear region. However, the striking feature of these derivatives is that although both oleic acid and linolenic acid translocated PKCα to the membrane, their fusion with the resveratrol moiety generated different response in that only **4**, not **2** translocated PKCα to the membrane. Our results clearly indicated that small changes in the chemical structure could lead to profound effect on the activation and translocation properties of PKCs. That the presence of different degree of unsaturation in the fatty acyl chain alters the properties of membrane, [65], [66] our results also imply that subtle ligand-protein-membrane interactions could dictate the activation mechanism of PKCs.

**Figure 14:**
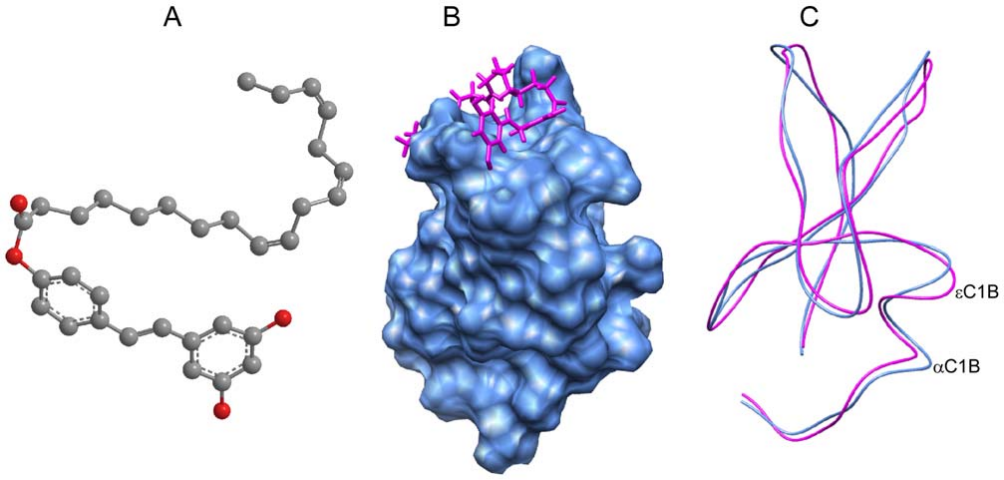
Molecular modeling. **A**) Energy minimized structure of **4. B**) Surface diagram of PKCαC1B (blue) docked with **4** (magenta). **C**) Overlaid structures of αC1B (blue) and εC1B (magenta). Structure of **4** was minimized using Chem3D pro 12.0.2. Molecular docking was done using sybyl 8.0. The protein structures were visualized using UCSF chimera 1.6.1.

The observation that resveratrol (**1**) inhibited TPA-induced PKC activation and competed with phorbol ester but not with the calcium, led Slater et al [50] propose that resveratrol binds at the phorbol ester binding site of PKCα. Our previous binding and modeling studies [54] on resveratrol derivatives also supported this prediction. However, addition of an aliphatic chain with unsaturation may alter its activity and protein binding mode. For example, chemical modification of the ultra-potent PKC activator phorbol esters turned them inhibitors of PKCα [67]–[69]. The ability of **4** in the activation of PKCα suggests that **4** could bind to its C1 domain. The energy minimized structure of **4** shown in Fig. 14A, revealed that the aliphatic chain formed a conformation that looked like a hook through which the molecule could anchor with the membrane. When the molecule was docked into the phorbol ester binding site of αC1B, the resveratrol moiety formed hydrogen bonds (the backbone NH of Gly-124 formed two hydrogen bonds with two oxygen atoms of the ester groups both at 2.86 Å, not shown) with the protein residues and the hydrocarbon chain capped the phorbol ester binding groove by interacting with the hydrophobic residues in present in the upper portion (Fig. 14B) making the surface hydrophobic. Because the structure of the εC1B has not been determined yet, a homology modeled structure is generated for the purpose of comparing it with αC1B. Superimposition of the structures revealed remarkable similarity between the overall structure of αC1B and εC1B (Fig. 14C), although several residues in the phorbol ester binding site were different. These residues are most probably responsible for different sensitivity of **4** for PKCα and PKCε. These differences in the residues in the C1B domains are also responsible for the difference in the binding affinity for phorbol ester, phorbol 12, 13-dibutyrate (PDBu) and DAG. For example, αC1B showed lower binding affinity for both phorbol ester and DAG than εC1B [70], [71] implicating different mechanism in PKCα and PKCε activation [71], [72]. Similarly, in spite of having conserved structure, δC1B binds to DAG/phorbol ester while Vav1 C1 does not [73].

That **4** acts on PKCα, which is Ca^+2^ sensitive, its interactions with the Ca^+2^ binding C2 domain cannot be completely ruled out. Several studies indicated that fatty acids bound to the phosphatidylserine (PS) [74]–[76] or calcium binding sites of C2 domain. This binding could also affect the binding of C2 domain with the isotype specific RACKs, [77] responsible for the PKC translocation.

That compound **4** shows reduced cytotoxicity, selectivity towards PKCα and also activated the downstream ERK1/2 either by the Ras →Raf →MEK1/2 →ERK1/2 or the Raf →MEK1/2 →ERK1/2 pathways, this compound can be used as a potential drug for disease states involving PKCα, such as cardiac contractility, atherogenesis, cancer and arterial thrombosis [29], [78], [79]. However further studies are required to ascertain if the selectivity of resveratrol and its derivatives is inherent to PKCα or dependent on the dynamic interactions of substrates, modulators and anchoring proteins present in a particular cell/tissue.

In summary, our results demonstrated that resveratrol moiety can be modified suitably as PKC selective modulators. Development of newer synthetic molecules around the resveratrol scaffold and studying their mechanism for isotype selectivity is warranted before any analog can be used as a drug candidate for a particular disease state.

## Materials and Methods

### General

Resveratrol, TPA and all other reagents were purchased from Sigma and used without further purification. Progress of chemical reaction was monitored through thin layer chromatography (TLC) on pre-coated glass plates (silica gel 60 F254, 0.25 mm thickness) purchased from EMD chemicals. ^1^H NMR and ^13^C NMR spectra were recorded on a QE-300 spectrometer. Unless otherwise specified, all NMR spectra were obtained in deuterated chloroform (CDCl_3_) and referenced to the residual solvent peak. Chemical shifts are reported in parts per million, and coupling constants in hertz (Hz). Multiplicities are reported as follows: s (singlet), d (doublet), t (triplet), m (multiplet) and br (broadened). Mass spectra were obtained on either a VG 70-S Nier Johnson or JEOL mass spectrometer.

### Synthesis of the resveratrol derivatives 2–5

**Figure 15:**
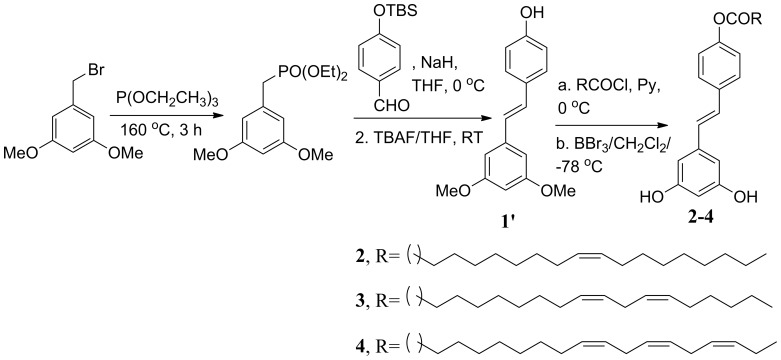
Synthetic scheme for compounds 2–4.

The synthetic procedure of **2–4** is outlined in Figure 15. Compound **1′** was prepared following our previously published methods [54] with minor modifications. For the synthesis of compounds **2–4,** compound **1′** was dissolved in anhydrous pyridine at 0°C and treated with the corresponding acid chloride (oleic acid chloride for **2**, linoleic acid chloride for **3** and linolenic acid chloride for **4**), which was prepared by the treatment of the corresponding acid (1 equivalent) in anhydrous dichloromethane with thionyl chloride (1.1 equivalent) and catalytic amount of DMF. Acid chloride was distilled under vacuum and used immediately for the next step. The reaction mixture was allowed to stir for 1 h at room temperature and then heated at 60 C for another 2 h. After cooling the mixture to room temperature, excess pyridine was removed in high vacuum. The compound was immediately used for the demethylation step. Demethylation was done by following the methods described earlier [54]. The compounds were purified by column chromatography (hexane: ethyl acetate: methanol, 60∶38∶2) and characterized by NMR spectroscopy and mass spectrometry.

4-((E)-3,5-dihydroxystyryl)phenyl oleate (**2**): Yield: 77%, ^1^H NMR (CDCl_3,_ 400 MHz) δ 7.47 (2H, d, *J* = 8.6 Hz), 7.06 (2H, d, *J* = 8.2 Hz), 7.00 (1H, d, *J* = 16.4 Hz), 6.88 (1H, d, *J* = 16.5 Hz), 6.55 (2H, d, *J* = 2 Hz), 6.26 (1H, t, *J* = 2.2 Hz), 5.33 (2H, m), 4.91 (2H, brs), 2.55 (2H, t, *J* = 7.8 Hz), 2.01 (4H, m), 1.75 (2H, m), 1.42–1.23 (20H, m), 0.86 (3H, t, *J* = 6.4 Hz); ^13^C NMR (CDCl_3_) δ 172.6, 157.0 (2C), 150.01, 139.9, 130.0, 129.8, 128.6 (2C), 128.2, 127.5, 121.9, 121.0 (2C), 106.2 (2C), 102.2, 34.2, 32.0, 29.7, 29.6 (2C), 29.4, 29.1 (2C), 28.9 (2C), 28.8 (2C), 26.8, 26.7, 13.1. ES-MS: 516 [M+Na].

(9Z,12Z)-4-((E)-3,5-dihydroxystyryl)phenyl octadeca-9,12-dienoate (**3**): Yield: 70%, ^1^H NMR (CDCl_3_) δ 7.46 (2H, d, *J* = 8.5 Hz), 7.06 (2H, d, *J* = 8.6 Hz), 7.00 (1H, d, *J* = 16.4 Hz), 6.88 (1H, d, *J* = 16.5 Hz), 6.51 (2H, d, *J* = 2.0 Hz), 6.25 (1H, t, *J* = 2.0 Hz), 5.36 (4H, m), 5.07 (2H, brs), 2.76 (2H, t, *J* = 5.04 Hz), 2.55 (2H, t, *J* = 6.0 Hz), 2.04 (4H, m), 1.75 (2H, m), 1.45–1.23 (14H, m), 0.88 (3H, t, *J* = 5.04 Hz); ^13^C NMR (CDCl_3_) δ 172.69, 157.06 (2C), 150.3, 139.9, 134.4, 130.3, 130.1, 128.5, 128.2 (2C), 128.1, 127.6 (2C), 121.9 (2C), 106.2 (2C), 102.4, 34.2, 31.8, 29.6, 29.4, 29.2 (2C), 29.1, 27.2 (2C), 25.7, 24.8, 22.2, 14.1; ES-MS: 514 [M+Na].

(9Z,12Z,15Z)-4-((E)-3,5-dihydroxystyryl)phenyl octadeca-9,12,15-trienoate (**4**): Yield: 68%, ^1^H NMR (CDCl_3_) δ 7.47 (2H, d, *J* = 8.2 Hz), 7.06 (2H, d, *J* = 8.7 Hz), 6.98 (1H, d, *J* = 16.2 Hz), 6.90 (1H, d, *J* = 16.0 Hz), 6.54 (2H, d, *J* = 2.0 Hz), 6.26 (1H, t, *J* = 2.0 Hz), 5.36 (6H, m), 4.95 (2H, brs), 2.80 (4H, t, *J* = 6.0 Hz), 2.55 (2H, t, *J* = 7.3 Hz), 2.01 (4H, m), 1.75 (2H, m), 1.42–1.34 (8H, m), 0.96 (3H, t, *J* = 7.0 Hz); ^13^C NMR (CDCl_3_) δ 172.4, 157.04 (2C), 150.37, 140.0, 134.5, 132.0, 130.3, 128.6, 128.3, 128.3, 128.2, 127.8 (2C), 127.5, 121.9 (2C), 106.2 (2C), 102.3, 34.5, 29.6, 29.2 (2C), 29.1, 27.2, 25.7 (2C), 25.6, 21.0, 14.3; ES-MS: 512 [M+Na].

The saturated analog (E)-5-(4-(hexadecyloxy)styryl)benzene-1,3-diol (**5**) was synthesized and characterized as described earlier [80].

### Spectral measurements

The UV-Vis absorption (Hitachi U-2910, Hitachi High Technologies America, Inc. Pleasanton, and CA) and fluorescence emission spectra (PTI-Quanta Master, Photon Technology, International, Inc., Birmingham, NJ) of resveratrol (**1**) and its derivatives (**2–5**) (1–10 µM) were recorded in different solvents at room temperature. Spectral maxima were determined from the fit of Gaussian function (Igor Pro 4, WaveMatrics, Inc, and Lake Oswego, OR).

### Cell cultures

CHO-K1 cells were maintained in humidified atmosphere (37°C, 5% CO2) and F12 medium supplemented with 2 mM glutamine, 10% fetal bovine serum (FBS) and 100 unit/ml antibiotics. Before treatment with compounds, cells were starved in without FBS media for 12 h at 60–70% confluency.

### Cytotoxicity assays

The cells were plated overnight in a 96-well plate (Corning, Corning, NY) at a density of 10^4^ cells per well. Cells were either treated with DMSO (1%), resveratrol or derivatives (1–100 µM) for 48 h. Cell viability was determined using Vybrant® MTT cell proliferation assay kit (Molecular Probes/Invitrogen, CA) as per the manufacturer's recommendations.

### Membrane fractionation and immunoblot

Compound-treated cells were washed and harvested in PBS. Cell lysis was carried out in lysis buffer (20 mM Tris-HCl, protease inhibitor, pH 7.4) with brief sonication (4 times, 5 second and 10% amplitude). Cell debris was removed by centrifuging the sample at 3500 rpm for 10 minute at 4°C. Protein concentration was measured using the BCA protein assay kit (Pierce, Rockford, IL). Cell lysate (25 µg protein/lane) was subjected to SDS-PAGE and immunoblot to detect protein expression. Cell lysate (200 µg protein/100 µl) was centrifuged at 40,000 rpm for 2 h at 4°C to separate out soluble (cytosolic) and pellet (membrane) fraction. Pellet fraction was incubated in lysis buffer (100 µl) containing 1% Triton X-100 for 1 h in ice, centrifuged at 40,000 rpm for 1 h and the supernatant was collected as the membrane fraction. The cytosolic and membrane fractions (30 µl) were subjected to SDS-PAGE (7%) and transferred to nitrocellulose membrane. Membranes were blocked with TBST (50 mM Tris-HCl, pH 7.4, 150 mM NaCl, 0.1% Tween 20) buffer containing 10 mg/ml BSA and then washed three times with the TBST buffer. Membranes were probed with primary antibody for overnight at 4°C and HRP-conjugated secondary antibody at room temperature for 1 h. Antibody dilutions were used as follows: anti-rabbit PKCα, 1∶500; anti-rabbit PKCε, 1∶500; anti-rabbit ERK, 1∶1000; anti-rabbit phopho-ERK, 1∶500; anti-rabbit β-actin, 1∶2000 and anti-rabbit HRP-conjugated, 1∶5000 (Cell Signaling, Danvers, MA). The blots were stripped and probed with β-actin and secondary antibody to check for equal loading. Protein bands were visualized using ECL (enhanced chemiluminescence) reagent (Pierce, Rockford, IL) and analyzed by AlphaImager® Gel Documentation system (Alpha Innotec, Santa Clara, CA).

### ERK1/2 activation

Activation of ERK1/2 was determined by treating the CHO-K1 cells either with 10 µM of the resveratrol derivatives for 24 h or 100 nM TPA for 1 h. ERK1/2 phosphorylation was measured by whole cell lysate immuno-blot analysis using phosphor-ERK1/2 specific antibody.

### Molecular modeling

The chemical structure of **4** was energy minimized using Chem3D pro 12.0.2 (Cambridgesoft) with 1000 iterations. Homology model for PKCεC1B and docking studies were performed using the methods described earlier [54], [80]. Protein structures were overlaid and visualized using UCSF Chimera 1.6.1.
